# A survey of the governance capacity of national public health associations to enhance population health

**DOI:** 10.1186/s12889-016-2935-y

**Published:** 2016-03-11

**Authors:** James Chauvin, Mahesh Shukla, James Rice, Laetitia Rispel

**Affiliations:** World Federation of Public Health Associations, c/o Institute of Global Health, University of Geneva, Biocampus - B304, chemin des Mines 9, 1202 Geneva, Switzerland; Management Sciences for Health, Medford, Massachusetts USA; Centre for Health Policy & Medical Research Council Health Policy Research Group, School of Public Health, Faculty of Health Sciences, University of the Witwatersrand, Johannesburg, South Africa

**Keywords:** Governance, Public health association, Capacity building, Organizational capacity, Organizational governance, World Federation

## Abstract

**Background:**

National public health associations (PHAs) are key partners with governments and communities to improve, protect and promote the public’s health. Governance and organizational capacity are among the key determinants of a PHA’s effectiveness as an advocate for appropriate public health policies and practice.

**Methods:**

During 2014, the World Federation of Public Health Associations (WFPHA) conducted an on-line survey of its 82 PHA members, to identify the state of organizational governance of national public health associations, as well as the factors that influence optimal organizational governance. The survey consisted of 13 questions and focused on the main elements of organizational governance: cultivating accountability; engaging stakeholders; setting shared direction; stewarding resources; and, continuous governance enhancement. Four questions included a qualitative open-ended response for additional comments. The survey data were analyzed using Microsoft Excel. The qualitative data was analyzed using thematic content analysis

**Results:**

Responses were received from 62 PHAs, constituting a 75.6 % response rate. The two most important factors that support governance effectiveness were a high degree of integrity and ethical behavior of the PHA’s leaders (77 %) and the competence of people serving on the PHA’s governing body (76 %). The lack of financial resources was considered as the most important factor that negatively affected organizational governance effectiveness (73 %). The lack of mentoring for future PHA leaders; ineffective or incompetent leadership; lack of understanding about good governance practices; and lack of accurate information for strategic planning were identified as factors influencing PHA governance effectiveness. Critical elements for PHA sustainability included diversity, gender-responsiveness and inclusive governance practices, and strategies to build the future generation of public health leaders.

**Conclusion:**

National PHA have a responsibility to put into place the practices and infrastructure that enhance organizational governance. This will enhance their ability to be effective advocates for policies and practices that enhance, protect and promote the public’s health. The WFPHA has an important role to play in providing the technical assistance and financial resources to assist PHAs in attaining and sustaining a higher level of governance capacity.

## Background

The concept of governance has generated a significant body of literature [1–11]. Governance is defined as “a collective process involving a group of stakeholders and it embraces the setting of strategic direction and objectives; making policies, laws, rules, regulations, or decisions; raising and deploying resources to accomplish the strategic goals and objectives; and ensuring that strategic goals and objectives are accomplished” [12]. In recent years, the global quest for the elimination of health inequalities [13] and for universal health coverage has put the spotlight on the importance of governance to achieve health and development goals, and to improve the performance of national health systems [1, 8, 10, 14–18]. Key elements of good governance for health include: evidence-informed decision-making; shared values of equity and social justice; transparent and inclusive processes; responsiveness to the needs of the people or beneficiaries; accountability by those who make or implement decisions; efficient and effective implementation of strategic objectives; and maintenance of organizational vitality [8, 18, 19]. Capacity for governance is an important determinant of good public health practice [20], as it enables improved population health outcomes and returns on health investment [21, 22]. Scholars have illustrated that improved governance is inversely related to under-5 child mortality rates [23]. However, insufficient attention has been given to the role of organizational governance, effectiveness and capacity in influencing health policy and practice in order to enhance population health [17, 18, 24].

The World Federation of Public Health Associations (WFPHA) was established in 1967 and is an international, non-governmental organization (NGO) comprising multi-disciplinary, national public health associations (PHAs) [25]. Public health refers to the “science and art of preventing disease, prolonging life and promoting health through organized efforts of society” [26], page xv. The WFPHA’s mission is to promote and protect public health throughout the world by: supporting the establishment and organizational development of PHAs and societies of public health; facilitating and supporting the exchange of information, knowledge and the transfer of skills and resources; and promoting and undertaking advocacy for public policies, programs and practices that will result in a healthy and productive world [25]. The WFPHA, being the only worldwide professional society representing and serving the field of public health, has seven categories of membership (Table 1).

**Table 1:** Categories of membership of the WFPHA

Category of membership	Brief description
Full Member	• A bone fide national public health association • Multi-disciplinary organisation enshrined in its constitution or articles of association • Meet all the criteria for Federation membership • Annual membership fee, according to category of country income • Full voting rights
Associate member	• National associations or organizations that meet some but not all of the WFPHA criteria • Membership reiewed every three years • Membership fees that is less than that of full members • Observer, non-voting status
Regional member	• Federations/networks of national public health associations or of Schools of Public Health of a geographic region • Do not pay membership fees • Observer, non-voting status
Sustaining member	• Organizations that do not meet the criteria for membership, but which endorse the principles of the Federation • Collaborate with Federation actively or through annual donations • non-voting but pay dues or contribute in-kind services and can attend meetings as observers
Individual Member	• Individuals who endorse the principles of the Federation and provide an active in-kind collaboration such as by participation in a Working Group or a monetary donation • Admission requires endorsement by their national PHA • No voting rights
Lifetime Member	• Individuals who have previously served as WFPHA Presidents or by judgment of the Governing Council having provided comparable significant service to the Federation may be appointed lifetime members • Non-voting observers • Exempt from annual membership fees
Honorary Member	• Recognition of individuals for a lifetime of commitment and a significant contribution to research, and/or education and/or service which has fundamentally advanced public health internationally • Honorary Membership is conferred for life • Exempt from annual membership fees

Source: http://wfpha.org/images/bylaws-and-co/Bylaws2015.pdf

In September 2014, the membership of the WFPHA consisted of 105 member national and regional public health associations, regional associations of schools of public health, and other non-governmental and governmental organizations and agencies. Of these, 82 were national PHAs located in 80 countries, with two countries (Bosnia & Herzegovina and the United Kingdom) each having two member associations, the former acknowledging the existence of distinct public health communities in the Federation of BiH and the Republika Srpska and the latter given the historical existence of two national organizations (the Royal Society of Public Health, a WFPHA founding member, and the Faculty of Public Health) representing that country’s public health community and interests.

The national PHAs that constitute the full members of the WFPHA vary greatly in size and capacity, and include younger more recent ones established in the past decade to the oldest PHA that is more than 100 years old. Nonetheless, PHAs regardless of size provide a forum for discussion and debate on a wide range of issues affecting the public’s health, bringing a broad spectrum of opinions or insights to frame the issues [27]. A national PHA facilitates evidence-based input from frontline health professionals and public health allies who wish to have a voice on issues that go beyond their everyday professional lives or for which there are challenges to expressing an opinion [27]. A PHA also provides an entry point for a politically non-partisan, independent voice to both the public as well as to key decision-makers. It plays a leadership role in increasing the visibility of public health as an essential component of a nation’s health system by galvanizing support through its capacity to convene people and organizations into partnerships for consensus-building, advocacy and action [27]. These PHAs have the potential to steer the development of solutions to complex social, economic and political determinants of health [18, 24].

In 2007, the WFPHA made a commitment to support national PHAs to improve their organizational infrastructure and capacity, including governance structures and processes, as part of its five-year strategic plan [28]. In a follow-up member survey conducted in 2009, 97 % of responding PHAs confirmed the presence of a governing body with 91 % providing some form of governance orientation to governing body members [28]. In the majority of cases, governing body members were elected to office. Nonetheless, many PHAs indicated governance challenges, which included limited financial sustainability and leadership deficiencies [28]. A subsequent WFPHA member survey, carried out in 2011, also identified organizational capacity building, including governance, as a priority need for PHAs in order to be effective advocacy organizations [29].

The 2013–2017 WFPHA Strategic Plan reaffirmed the importance of improving organizational governance structures and processes, infrastructure and capacity as a key goal [30]. However, the WFPHA Federation lacked more detailed information on: PHA governance capacity, challenges of governance structure and processes, and actions needed to enhance their organizational governance and capacity. Hence the goals of the 2014 survey were to explore several issues: PHA knowledge of the Federation’s revised strategic plan; the state of organizational governance of national PHAs; the factors that influence organizational governance; and what PHAs felt the WFPHA could do to help improve their organizational capacity and effectiveness.

This article focuses on the results and interpretation of the 2014 survey’s elements related to organizational governance effectiveness.

## Methods

### Ethics statement

The 2014 survey was part of ongoing assessments conducted by the WFPHA to determine what support it should provide to member associations to enable them to fulfill their mandates as national PHAs. The survey was one of the key activities of the 2013–2017 WFPHA Strategic Plan, and was approved by the Federation’s Governing Council. Following approval by the Governing Council, a cover letter was sent to the representative of each national PHA, obtained from the WFPHA database. The cover letter outlined the purpose of the survey, the voluntary nature of participation, and an electronic link to the survey was provided. The representative of the PHA was assured of the confidential nature of the survey, and that the survey results would be reported cumulatively such that no single PHA could be identified. The participating PHAs were also informed that the survey results would be put in the public domain and made available to all member organizations. The study received ethics approval from the Health Research Ethics Committee of the University of the Witwatersrand (Johannesburg, South Africa).

### Study design

The population of interest was the full members of the WFPHA (Table 1), which consisted of 82 national PHAs located in 80 countries, as explained above.

During 2014, an on-line, cross-sectional survey was conducted using Survey Monkey. The survey tool was based on a governance framework developed by Management Sciences for Health (MSH), a USA-based international development NGO implementing a USAID-sponsored Leadership, Management and Governance project [12, 31]. The key elements of this governance framework are: cultivating accountability; engaging stakeholders; setting shared direction; stewarding resources; and continuous governance enhancement [12, 31]. The questions were adapted to take account of the WFPHA’s strategic plan and the two previous surveys conducted in 2009 and 2011.

The survey tool consisted of 13 over-arching questions. Of these, three related to the perceived role and essential functions of a PHA and the issues that PHAs perceive as priorities for action, seven dealt with various aspects of organizational governance, and two with PHA members’ knowledge about the WFPHA. Each of these questions consisted of multiple items, measured on a 5-point Likert scale, wherein 1 indicated ‘very little importance/support’ or ‘very little application/practice’ and 5 ‘critical importance/very big support’ or ‘fully applied/practised’.

The survey tool included four open-ended questions for additional comments. These questions provided PHAs with the opportunity to expand on: the perceived roles and responsibilities of a PHA; the factors supporting governance effectiveness; factors constraining organizational governance; and what the WFPHA could do to help improve the individual PHA’s organizational capacity and effectiveness. The questionnaire was produced in three languages, English, French and Spanish, and was pretested in the three languages to ensure consistency and to ensure the same meaning when back-translated into English. The survey questionnaire is available upon written request from the WFPHA.

### Data collection and analysis

A cover letter with the pre-announcement about the survey was sent by email in early September 2014 to the 82 Full Member PHAs. The initial invitation to complete the survey was sent 10 days later in a personalized email addressed to the representatives of each of these PHAs. Two follow-up reminder emails were sent to all non-respondents. The on-line survey was closed on 19 December 2014.

The closed-ended questions were analyzed using Microsoft Excel. Frequency tabulations were done to describe the responses to the governance questions. The responses to the open-ended questions were analysed using thematic content analysis [32]. The first step in the analysis was to look at the words and phrases and without preconceived notions or classification. To ensure reliability, two of the authors participated in the development of the themes by reading the responses independently in order to establish inter-coder agreement [32, 33]. Once the initial analysis was completed, the two discussed the themes generated independently, and reached agreement on the themes.

## Results

We obtained an overall response rate of 75.6 % or 62 PHAs in 61 countries. The response rate by WFPHA geographic region is shown in Table 2, and ranged from a low of 51.3 % for Europe and Central Asia, to 100 % for four of the regions.

**Table 2:** WFPHA survey response rate by region

Region (as defined by WFPHA)	Explanatory note	Response rate
Africa	PHAs in 18 countries in continental Africa, excluding Egypt	16/18 = 88.9 %
Americas (North, Central, South and Caribbean)	PHAs in 10 countries	10/10 = 100 %
Asia-Pacific (including Oceania)	PHAs in 10 countries	10/10 = 100 %
Europe and Central Asia (including Turkey and Israel)	PHAs in 37 countries	19/37 PHAs = 51.3 %
Middle East (Egypt, Eastern Mediterranean/Arab Peninsula/Gulf States, Iran and Afghanistan)	PHAs in 4 countries	4/4 PHAs = 100 %
South Asia (excluding Afghanistan)	PHAs in 3 countries	3/3 PHAs = 100 %

The majority of PHAs (*n* = 45; 72 %) responded using the English version, 17.7 % (*n* = 11) responded using the French version, and 6 (10 %) responded using the Spanish version.

### Perceived importance of governance for health

Table 3 shows PHA responses to the 11 pre-defined functions for a PHA. As can be seen, increasing awareness among decision-makers and the public about “what is public health” scored the highest, with 87 % of PHAs indicating this factor as very important or critically important. The issue scoring the lowest importance for a PHA to focus on was “link people to health services they need”.

**Table 3:** PHAs perceptions of relative importance of public health focus areas

Degree of importance of different public health focus areas	n	Very or critically important (% respondents)
Increase awareness among decision-makers and the public about public health	54	87 %
Mobilize partnerships and action to identify and solve health problems	50	81 %
Inform, educate, and empower people about health issues	49	79 %
Conduct research to develop new insights and innovative solutions to public health problems	41	66 %
Analyze and investigate health problems and health hazards	40	65 %
Maintain a competent public health care workforce	40	65 %
Develop policies and plans that support individual and community health efforts	38	61 %
Evaluate effectiveness, accessibility, and quality of population-based health services	37	60 %
Monitor health status to identify and solve health problems	35	56 %
Enforce laws and regulations that protect health and ensure safety	33	53 %
Link people to health services they need	27	44 %

In the open-ended responses received, the majority of PHAs indicated that policy and practice advocacy and influencing health-related policy and practices, and creating a strong and independent civil society voice for public health were important priorities. These comments are reflected in the excerpts below:

- “Plaider en faveur de systèmes de santé publique efficaces dans les pays et la réduction des inégalités sociales en matière de santé [Advocate for effective health systems and for a reduction in health-related social inequities]” (PHA1)
- “Advocating for the adoption of healthy public policy which address the social and ecological determinants of health” (PHA2)
- “Public health activism with raising awareness about major health problems and mobilization of citizens for active participation in health promotion” (PHA3)

PHA respondents also identified the role of PHAs as a ‘catalyst’ (creating opportunities for better health and improved health equity), playing the role of ‘convener’ (developing and nurturing inter-sectoral partnerships for consultation and action) and being a ‘collaborator’ by working with other health and non-health actors to find and help put into place solutions to issues affecting the public’s health.

### Factors constraining organizational governance and governance effectiveness

Governance effectiveness in the context of a PHA is the degree to which its governing body is successful in realizing its strategic direction and achieving its mission. In the survey, 73 % of PHAs identified the lack of financial resources from national and international donors as a very or critically important factor that constrains the capacity of a PHA to be influential and effective (Table 4). Around one third of PHAs identified the lack of competent persons serving on the governance body (34 %); lack of mentoring of future PHA leaders (32 %); and lack of accurate information for strategic planning (31 %) as other constraining factors.

**Table 4:** Factors that constraint governance effectiveness of PHAs

Constraining factor	n	Big or very big constraint (% respondents)
Lack of financial resources for the PHA	45	73 %
Lack of competent persons serving on the governing body	21	34 %
Lack of mentoring for future PHA leaders	20	32 %
Lack of accurate information for good strategic planning	19	31 %
Lack of understanding about practices of good governance	18	29 %
Lack of or ineffective leadership	17	27 %
Lack of transparency and accountability	15	24 %

**Table 5:** Factors that support PHA governance effectiveness

Supporting factor	n	Big or very big support (% respondents)
High degree of integrity and ethical behavior of association leaders	48	77 %
Competent persons serving in the governing body	47	76 %
High degree of transparency and accountability in decision-making	42	68 %
Free and informed media relations	39	63 %
Clarity about how to practice good governance	37	60 %
Accurate information for planning and evaluating the work of the PHA	35	56 %
Broad-based engagement of stakeholders in decision-making process	34	55 %
Government support (moral and financial)	34	55 %
Sufficient financial resources	33	53 %
Availability of staff to implement governance decisions	30	48 %
High degree of donor interest	22	35 %

The inability of voluntary members of PHAs to dedicate time and lack of interest among younger PHA members for organizational activities emerged as issues in the qualitative responses, shown by the excerpts below:

- “Peu ou pas de jeunes cadres s'intéressant aux activités des ASP [Few or no young professionals interested in the PHA’s activities]” (PHA4)
- “Falta de tiempo del cuerpo directivo para dedicarse a la tarea de gobernar la asociación [Lack of time devoted by the governing body to the task of governing the association]” (PHA5)
- “Lack of time for the persons involved, which have their full time job and work for the association on voluntary basis” (PHA6)

### Factors supporting governance effectiveness

Of the 11 predefined factors that support governance effectiveness, the two most important factors identified by respondents were the high degree of integrity and ethical behavior of the PHA’s leaders (77 %) and the competence of people serving on the PHA’s governing body (76 %) (Table 5).

The qualitative comments, as exemplified by the examples below, suggest that the availability of financial resources in support of the PHA’s organizational functions, dedicated staff and a democratic political environment facilitate the governance effectiveness of PHAs.

- “Practices including gender-responsive governance.” (PHA7)
- “A good strategic plan backed up by resources.” (PHA8)
- “In all of these factors there can always be more - eg money, staff, involvement.” (PHA9)
- “We need to expand our staff, but we have financial constraints to do it.” (PHA10)

### Good governance practices

Table 6 shows the extent to which the PHAs implement or practise good organisational governance in the areas of: cultivating accountability, engaging stakeholders, setting shared strategic direction, stewarding resources, and activities related to continuous governance enhancement.

**Table 6:** Good practice governance applied fully or almost fully by PHAs

Practice of good governance	n	Applied almost fully or fully (% respondents)
Cultivating accountability		
Fosters internal accountability in the association	46	74 %
Shares information	44	71 %
Cultivates personal and collective accountability	40	65 %
Provides effective financial and quality oversight	36	58 %
Nurtures accountability of the PHA to its external stakeholders	35	56 %
Measures performance	31	50 %
Engaging stakeholders		
Engages with PHA members	44	71 %
Promotes equity	42	68 %
Builds relationship of trust with PHA’s stakeholders	41	66 %
Practises gender-responsive governance	37	60 %
Engages PHA’s diverse stakeholders	36	58 %
Collaborates with other sectors	36	58 %
Engages with health workers and providers	33	53 %
Engages with communities	31	50 %
Setting shared strategic direction		
Defines a strategic plan for the PHA	44	71 %
Establishes shared vision among key stakeholders	42	68 %
Builds leadership capacity within the PHA	38	61 %
Monitors progress in implementing the strategic plan	34	55 %
Stewarding resources		
Practices ethical and moral integrity	51	82 %
Eradicates corruption	50	81 %
Wisely uses resources	46	74 %
Pursues efficiency and sustainability	43	69 %
Uses information, evidence and technology in governance decision-making	40	65 %
Mobilizes resources	30	48 %
Continuous governance enhancement		
Builds diversity in the association	45	73 %
Cultivates governance competencies	38	61 %
Organizes governance orientation and continuous governance education	31	50 %
Performs regular governance assessments	22	35 %

As can be seen from Table 6, there was great variation in the extent to which PHAs indicated that these good practices were in place for each of the categories contained in the conceptual framework, with scores of 70 % or higher obtained for: fostering internal accountability (74 %); sharing information (71 %); engagement with PHA members (71 %); defining a strategic plan for the PHA (71 %);practicing ethical and moral integrity (82 %); eradicating corruption (81 %); wise resource utilisation (74 %) and building diversity in the organization (73 %). The lowest score was obtained for performing regular governance assessments (35 %).

PHAs indicated that critical elements for sustainability included diversity, gender-responsiveness and inclusive governance practices, and strategies to build the future generation of public health leaders.

### The role of the WFPHA in building PHA governance and organizational capacity

The survey elicited 48 responses (84.2 % of respondents) to the survey’s open-ended final question about the PHAs major expectations of the WFPHA.

PHAs requested the WFPHA to assist in their organizational capacity building by providing advice and facilitating and enhancing access to sources of funding/technical support to PHAs located in low- and middle-income countries. They also suggested twinning newer and organizationally weaker PHAs with more mature and organizationally well-developed PHAs. They also requested the WFPHA to organize and help host skills and competency building workshops on various issues (e.g., policy development and advocacy, PHA governance and management, revenue generation and financial accounting, identifying and nurturing future PHA leadership.

## Discussion

In concert with the 2013–2017 WFPHA Strategic Plan that highlights the importance of effective organizational governance, [30], this survey aimed to determine the views of the WFPHA members on factors that influence governance effectiveness of PHAs, the extent to which PHAs adhere to good governance practices, and the role of the WFPHA in enhancing PHAs’ organizational capacity and effectiveness.

Three quarters of PHAs (75.6 %) responded to the survey, indicating the importance accorded by national PHAs to the issue of effective governance, and their desire to engage with and support the work of the Federation. In four of Federation’s regions, 100 % response rates were obtained from PHAs. The relatively low response rate of PHAs in Europe and central Asia could be an indication of the relative well-resourced nature of these PHAs, requiring little support from the WFPHA for governance capacity development or related activities. It could also be because of the time of year that the survey was conducted (between September and December 2014, when several European PHAs were holding their annual conferences and membership meetings).

The high priority accorded by responding PHAs to educating decision-makers and the general public about public health, partnerships, and taking action to identify and solve health problems is not surprising, as these activities constitute the core of public health [26]. These issues were also among those identified in a 2011 literature review and interviews with key informants in the USA as the key functions of public health governance [18].

In this survey, the PHAs reported the lack of financial resources as one of the major factors that constrains their capacity to be influential and effective (Table 4). Sufficient funding in turn affects the ability of PHAs to appoint and retain full-time staff, and to engage in major advocacy activities, especially those requiring finances. The survey’s results are consistent with the lessons learned through the Strengthening of Public Health Associations (SOPHA) Program, an initiative implemented and managed by the Canadian Public Health Association between 1985 and 2011 [34]. The SOPHA program provided funding and technical assistance to PHAs in low- and middle-income countries to enhance their organizational capacity and effectiveness [34]. A critical factor to the success of a PHA, identified through the SOPHA Program [34], was the presence of an adequately staffed and resourced secretariat, with one of its functions being to service the PHA’s governance. Although volunteerism is alive and well within PHAs, Chauvin et al. [27] have pointed out that volunteerism is important, but it is not a panacea. Complete reliance on volunteerism to drive and maintain a PHA is insufficient for organizational sustainability. Furthermore, there are limitations to the activities that can be done by voluntary PHA members. Full time, paid staff members and a well-resourced secretariat enable the growth and effectiveness of PHAs as these staff members can assist with monitoring and follow up of strategic goals, ensure implementation of these goals, thus enhancing accountability to PHA members. The constraining aspect of the lack of finances was also found in a study in Afghanistan that explored the use of community scorecards to enhance accountability for people-centred health care [11]. The Afghanistan study found that lack of funding influenced the ability to deliver on targets for delivery which in turn affected accountability and governance [11].

PHAs identified ethical behavior of the PHA’s leaders (77 %) and the competence of people serving on the PHA’s governing body (76 %) as the main factors supporting governance effectiveness. The SOPHA program also found that the critical enabling factors for PHAs effectiveness included: leadership and stewardship; accountability for making and implementing decisions; engaging with its members and other critical stakeholders, and efficient and effective implementation of the PHA’s strategic objectives [34].

It is encouraging that 82 % of PHAs reported that they practise ethical and moral integrity and 81 % indicated that they take steps to eradicate corruption. This is important as a national PHA facilitates independent civil society advocacy for policies and practices that prevent injury and disease, and protect and promote health and health equity. In some countries, PHAs are the only citizen ‘voice’. Citizen involvement and advocacy is one of the key interventions to combat corruption highlighted by the a recent South African study [35]. At the same time, the study found that there was great variation in the extent to which PHAs indicated that these good practices were in place for each of the categories contained in the conceptual framework. Only 35 % of PHAs reported that they perform regular governance assessments, which contribute to a well-functioning and effective PHA [36]. Through ‘good’ governance, organizational leaders have the capacity to set direction for the organization, protect its mission, ensure that tasks are completed, support resource development and ensure a regular succession of new leaders [37].

Nonetheless, the survey’s results confirm that most PHAs recognize the role their organizations play in public health, and the importance of ensuring good governance and a sustainable association (in terms of funding and staffing). Weaknesses in organizational governance and the lack of good governance practices will influence PHAs’ ability to set direction, stick to a defined mission, carry out its strategic tasks or identify and nurture future leaders. The PHA respondents, through the survey, acknowledge these gaps and state clearly their desire for technical mentoring and assistance to improve their organizational governance capacity.

PHAs recognized continuous governance enhancement as an important component of investing in the future strength of the PHA, sighting it as a means of building diversity to enhance organizational strength. One means of achieving this is through the nurturing and mentoring of leadership both within the governing body and of identifying and nurturing younger PHA members as a critical element for PHA advocacy effectiveness and sustainability. This includes applying and monitoring gender-responsive and inclusive governance practices that engage with and involve actively vulnerable populations.

The study suffered from several limitations. The findings are based on self-reported information from PHAs, and there may have been some social desirability bias [38]. The cross-sectional design means that the study reflects the views of participating PHAs at a point in time. Nonetheless, the study makes an important contribution to the discourse on organizational governance and the factors influencing the effectiveness of such governance.

Several PHAs, such as those in Canada, the United States, South Africa, Ethiopia, Australia, Brazil and some European countries, offer opportunities through membership within their governance body and leadership training/internships as a means of engaging younger members for the long term. The WFPHA, for its part, launched in 2013 the pilot WFPHA Fellowship program, wherein representatives from organizationally weaker PHAs are imbedded within a more organizationally mature PHA to learn first-hand how it operates, both in terms of governance and operations. The WFPHA also began in 2013 to offer skills-building workshops on policy development and advocacy. The impact of these pilot initiatives will be assessed and revised over the next three years to contribute to enhancing governance and leadership, in line with the goal to promote and support the advancement of strong member associations within the Federation’s 2013–2017 Strategic Plan.

Good governance is not static; achieving good governance over the long term requires monitoring and assessing the performance of an organization’s governance structure and processes. Those who govern must make individual and collective commitment to enhance the strategies, structures, and style of the governing practices continuously. The survey findings have provided much food for thought to the Federation’s Governing Council, which will need to develop creative strategies to support PHAs and enhance their governance capacity for improved population health.

## Conclusion

National PHAs have a responsibility to put into place the practices and infrastructures that enhance organizational governance. The survey confirmed that organizational governance is both perceived as being critical to and has an impact on the effectiveness of PHAs in their role as advocates to improve, protect and promote the public’s health. Several factors that limit effective governance, such as the role and effectiveness of volunteers, mentorship and leadership training and cultivating a culture of good governance and accountability, can be dealt with internally. Others, such as building alliances, engaging stakeholders and creating solidarity and movement forward on global public health issues, will require external collaboration and partnerships. The WFPHA has an important role to play in providing the technical assistance and financial resources to assist PHAs in attaining and sustaining a higher level of governance capacity.

Organizational governance capacity building and continuous governance monitoring and enhancement should be incorporated as part of the global efforts to achieve a more egalitarian and socially just world.
